# Why some tumours trigger neovascularisation and others don’t: the story thus far

**DOI:** 10.1186/s40880-016-0082-6

**Published:** 2016-02-12

**Authors:** Omanma Adighibe, Russell D. Leek, Marta Fernandez-Mercado, Jiangting Hu, Cameron Snell, Kevin C. Gatter, Adrian L. Harris, Francesco Pezzella

**Affiliations:** Radcliffe Department of Medicine, Nuffield Division of Laboratory Science, John Radcliffe Hospital, University of Oxford, Oxford, OX3 9DU UK; Radcliffe Department of Medicine, Leukaemia and Lymphoma Research Molecular Haematology Unit, Nuffield Division of Laboratory Science, John Radcliffe Hospital, Oxford, OX3 9DU UK; Biodonostia Research Institute, Oncology Area, San Sebastian, Spain; Department of Medical Oncology, Molecular Oncology Laboratories, Weatherall Institute of Molecular Medicine, John Radcliffe Hospital, Oxford, OX3 9DU UK

**Keywords:** Cancer, Angiogenesis, Hypoxia, Blood vessels

## Abstract

**Background:**

Angiogenesis is not essential for tumours to develop and expand, as cancer can also grow in a non-angiogenic fashion, but why this type of growth occurs is unknown. Surprisingly, our data from mRNA transcription profiling did not show any differences in the classical angiogenic pathways, but differences were observed in mitochondrial metabolic pathways, suggesting a key role for metabolic reprogramming. We then validated these results with mRNA profiling by investigating differential protein expression via immunohistochemistry in angiogenic and non-angiogenic non-small cell lung cancers (NSCLCs).

**Methods:**

Immunohistochemical staining for 35 angiogenesis- and hypoxia-related biomarkers were performed on a collection of 194 angiogenic and 73 non-angiogenic NSCLCs arranged on tissue microarrays. Sequencing of P53 was performed with frozen tissue samples of NSCLC.

**Results:**

The non-angiogenic tumours were distinguished from the angiogenic ones by having higher levels of proteins associated with ephrin pathways, mitochondria, cell biogenesis, and hypoxia-inducible factor 1 (HIF1) regulation by oxygen and transcription of HIF-controlled genes but lower levels of proteins involved in the stroma, cell–cell signaling and adhesion, integrins, and Delta-Notch and epidermal growth factor (EGF)-related signaling. However, proteins classically associated with angiogenesis were present in both types of tumours at very comparable levels. Cytoplasmic expression of P53 was strongly associated with non-angiogenic tumours. A pilot investigation showed that P53 mutations were observed in 32.0% of angiogenic cases but in 71.4% of non-angiogenic tumours.

**Conclusions:**

Our observations thus far indicate that both angiogenic and non-angiogenic tumours experience hypoxia/HIF and vascular endothelial growth factor (VEGF) pathway protein expression in a comparable fashion. However, angiogenesis does not ensue in the non-angiogenic tumours. Surprisingly, metabolic reprogramming seems to distinguish these two types of neoplastic growth. On the basis of these results, we raise the hypothesis that in some, but not in all cases, initial tissue remodeling and/or inflammation could be one of the secondary steps necessary to trigger angiogenesis. In the non-angiogenic tumours, in which neovascularisation fails to occur, HIF pathway activation could be the driving force toward metabolic reprogramming.

**Electronic supplementary material:**

The online version of this article (doi:10.1186/s40880-016-0082-6) contains supplementary material, which is available to authorized users.

## Background

In 1927, Otto Warburg described what would be called the “Warburg effect,” in which tumour cells exhibited characteristic changes in metabolism, particularly the use of glycolysis rather than oxidative phosphorylation, despite the presence of adequate amounts of oxygen [1, 2]. Warburg believed that this process was the actual cause of neoplastic transformation [3].

Tumour development is now known to be driven by genetic damage. However, mutations in some metabolic enzymes, such as succinate dehydrogenase (SDH) and fumaratehydratase (FH), both parts of the tricarboxylic acid (TCA) cycle, can drive neoplastic transformation, and intermediate products of metabolism can also promote neoplastic progression [4].

Proliferating cancer cells have a high energy requirement to maintain homeostatic cellular processes. The shift in energy production to aerobic glycolysis, while allowing for more rapid production of adenosine triphosphate (ATP), yields far less energy than oxidative phosphorylation: there are two net molecules of ATP per glucose molecule in glycolysis versus 36 molecules of ATP via oxidative phosphorylation [5].

The reasons for the glycolytic energy dependence of proliferating tumour cells are still being debated. Initially, it was believed that the mitochondria in tumours were intrinsically defective. However, it was determined that tumour mitochondria are actually functional, retaining the capacity for oxidative phosphorylation and consuming oxygen at similar rates to normal tissues [6], although it should be appreciated that a degree of variability in mitochondrial activities exists across different neoplasms. Alternatively, high rates of glycolysis might be co-selected with factors that promote the expression of hypoxia-related genes (such as those required for angiogenesis) as an oxygen-independent energy source. Finally, increased intermediate products of glycolysis can easily be shunted into the biosynthetic pathways required for serine and nucleotide synthesis [7].

According to Folkman’s original theory [8], the onset of hypoxia in tumour triggers angiogenesis, which in turn is essential for supplying neoplastic cells with nutrients and oxygen and evacuating metabolic waste and carbon dioxide. The best understood hypoxia signaling mechanism is the stabilization and post-transcription activation of the hypoxia-inducible factor (HIF) proteins, which lead to the activation of many different pathways, including the vascular endothelial growth factor (VEGF) pathway. The VEGF pathway prompts and supports neoangiogenesis and glycolysis. Hypoxia-inducible pathway activation also has other effects, which include reducing the activity of mammalian target of rapamycin (mTOR), which in turn can reignite autophagy and promote survival under stress [9].

HIF is a heterodimer of an alpha subunit that is unstable in normoxia and a constitutively present and stable beta subunit. The hypoxia activation of HIF causes the heterodimer to bind to DNA at specific locations, called hypoxic response elements (HREs), eliciting the transcriptional up-regulation of genes required to respond appropriately to hypoxia [9].

In addition to triggering the VEGF pathway, the ubiquitously expressed HIF1 isoform promotes the transcription of glucose transporter 1 (GLUT1), which activates glucose transport inside the cell, lactate dehydrogenase-A (LDH-A), which is involved in the glycolytic pathway, erythropoietin (EPO), which enhances erythropoiesis, and nitric oxide synthase (NOS), which promotes angiogenesis and vasodilatation [9].

HIF1 also prevents the entry of pyruvate into the TCA cycle by inducing the expression of pyruvate dehydrogenase kinase 1 (PDK1), thus altering the expressed isoform of cytochrome *c* and inhibiting mitochondrial biogenesis. This process causes reduced levels of oxygen consumption and a shift away from oxidative phosphorylation. Interestingly, HIF1 can also be activated under normoxic conditions by a variety of oncogenic pathways, such as phosphatidylinositol-4,5-bisphosphate 3-kinase catalytic subunit alpha (PIK3CA), and by mutations in von Hippel-Lindau tumour suppressor (VHL), SDH, and FH [10].

In the classic angiogenic pathway, VEGF binds to VEGF receptor 2 (VEGFR2) on endothelial cells, increasing the expression of the Notch ligand Delta-like 4 (DLL4) on the same cells. DLL4 then binds to its receptor Notch on the adjacent endothelium. Further expression of VEGFR2 and VEGFR1, as well as a smaller amount of VEGFR3, then follows, leading to triggering/amplification of the downstream phospholipase C familyγ (PLCγ)–protein kinase C (PKC)–Raf kinase–MAP kinase-ERK kinase (MEK)–mitogen-activated protein kinase (MAPK) pathway, concomitantly prompting cell proliferation and cell survival throughout the phosphoinositide 3-kinase (PI3 K)/protein kinase B (AKT) pathway [11].

The switch to glycolysis in neoplasia was, according to Warburg, irreversible [3], yet a more complex picture has emerged over the last decade. There have been observed instances in which oxidative phosphorylation predominates during neoplastic transformation [12]. This variation between OxPhos and glycolysisin cancer cells has been increasingly linked to specific disturbances in cell signaling pathways [13].

Additionally, tumours of the same genetic lineage can develop different metabolic adaptations depending on the host tissue from which they arise, suggesting that the stromal environment might play a crucial role in shaping the metabolic profile [14]. The different molecular mechanisms being postulated to explain this variability of the Warburg effect include the following: inhibition of pyruvate dehydrogenase (PDH) by PDK1, reduction of mitochondrial biogenesis and inhibition of oxidative phosphorylation, both are caused by P53 inactivation and mutations [15].

Warburg raised two important issues: first, how tumour cells are supplied with glucose; and second, how they are supplied with oxygen [1]. Folkman’s work addressed the latter question with the hypothesis that tumour growth is strictly angiogenesis-dependent [16]. The work undertaken to test this hypothesis led to the inclusion of “angiogenesis” as one of the hallmarks of cancer [8].

Although there is strong evidence that angiogenesis frequently occurs in cancer, we also now know that this event does not always occur. Indeed, some tumours, called “non-angiogenic tumours,” can grow without triggering new vessel formation by co-opting preexisting vessels [17, 18].

Non-angiogenic growth was first identified by histology in primary and metastatic lung carcinomas because neoplastic cells filled the alveolar spaces, co-opting the pre-existing capillary network and exhibiting a characteristic “chicken-wire” appearance [17]. A gene expression signature for non-angiogenic non-small cell lung cancer (NSCLC) was published in 2005 [19]. Surprisingly, rather than the classic angiogenesis-related genes, the differentially expressed genes were involved in mitochondrial metabolism, transcription, protein synthesis, and the cell cycle. Lack of differential mRNA expression between tumour phenotypes was noted for genes classically associated with hypoxia and angiogenesis. This result suggested that the response to hypoxia does not necessarily trigger neovascularisation, as would be observed in angiogenic tumours, but could actually be dependent on the genetic background of neoplastic cells, and in some instances, it could lead to metabolic reprogramming [19]. We therefore postulated that the degree to which a tumour will rely on angiogenic or non-angiogenic growth could be associated with a variety of events, including hypoxia, pseudo-hypoxia, and metabolic re-programming.

In the first part of the present study, we investigated whether there were truly no differences in the expression of hypoxia-and angiogenesis-related proteins between angiogenic and non-angiogenic tumours, as suggested by mRNA profiling. We also investigated the degree of these proteins expression and the expression of some mitochondrial biogenesis proteins via immunohistochemistry. Notably high cytoplasmic P53 expression in non-angiogenic tumours, compared to angiogenic tumours, was found after completing the first part of this study. We therefore performed a second investigation, in which we examined and sequenced the *p53* gene in these tumours.

## Methods

### Tissue specimens

Clinical specimens of NSCLCs were obtained from a series of consecutive patients who underwent surgical treatment at the John Radcliffe Hospital, Oxford, UK. This collection had ethical committee approval (study number C02.216—The pathophysiology of human neoplasia). The tissues were formalin-fixed and paraffin-embedded. Tissue microarrays were constructed using the Beecher Instrument MTA-1 manual arrayer (Beecher Instruments, Inc., Sun Prairie, WI, USA). Up to four suitable areas of appropriate tumour were chosen from a slide stained with hematoxylin and eosin (H&E), avoiding areas of necrosis.

### Immunohistochemical staining of tissue sections

The 4-μm sections were cut from paraffin blocks and mounted on glass histology slides. Non-specific protein binding was blocked by incubating with 2.5% normal horse serum (Vector Laboratories, Burlingame, CA, USA), and the primary antibody was then applied. Details of all of the antibodies used are presented in Table 1. Substitution of the primary antibody with phosphate-buffered saline (PBS) served as a negative control. The slides were then counterstained with hematoxylin for 20 s.

**Table 1:** Antibodies used for immunohistochemistry

Antigen	Clone	Source
HIF1	ESEE122	Abcam (Cambridge, UK)
HIF2	EP109b	NDCLS (University of Oxford, John Radcliffe Hospital, Oxford, UK)
CA9	M75	BioScience Slovakia s.r.o. (Bratislava, Slovakia)
VEGFA	VG-1	NDCLS
TYMP	PGF44	NDCLS
KDR	FLK1(A3)	Santa Cruz Biotechnology, Inc. (Dallas, TX, USA)
KDRp34	34a	NDCLS
FIH	FIH162c/D6	NDCLS
PHD1	PHD112/G	NDCLS
PHD2	366G/76	NDCLS
PHD3	EG188c	NDCLS
DLL4	D4/37	NDCLS
TSP1	8A6B-TSP-1	Leica Microsystems (UK) Ltd. and Novocastra Reagents (Milton Keynes, UK)
CXCR4	MAB 172	R&D System (Minneapolis, MN, USA)
EPHB2	AF496	R&D System
EPHB3	R&D AF432	R&D System
EPHB4	R&D AF446	R&D System
SOD1	30F11	Novocastra
BCL2	124	Dako (Cambridgeshire, UK)
FOS	Polyclonal 27436	Abcam
EGF	EGF 10	Abcam
EGFR	F4	Abcam
BNIP3	Ana40	Sigma-Aldrich Company Ltd. (Dorset, UK)
P53	DO7	Dako
PI3	Ab 40755	Abcam
SP1	SP1 polyclonal	Abcam
STAT3	E121-21	Abcam
LON	20H1	NDCLS
MEF2D	MEF2D polyclonal	Abcam
JMY	HMY117A	NDCLS
TRAP1	TR-1A	LabVision (TorsbySjöväg, Värmdö, Sweden)
GST	GST3/GST pi	Abcam
NCAM	IB6	Novocastra
CHGA	FLEX polyclonal	Dako
SYP	299	Novocastra

*HIF* hypoxia-inducible factor; *CA9* carbonic anhydrase 9; *VEGFA* vascular endothelial growth factor A; *TYMP* thymidine phosphorylase; *KDR* vascular endothelial growth factor receptor 2; *KDRp34* vascular endothelial growth factor p34; *FIH* factor-inhibiting HIF; *PHD* prolyl hydroxylase dehydrogenase; *DLL4* Delta-like 4; *TSP1* thymidine phosphorylase 1; *CXCR4* chemokine (C-X-C motif) receptor 4; *EPH* ephrin; *SOD1* superoxide dismutase 1; *BCL2* B cell lymphoma 2; *FOS* FBJ murine osteosarcoma viral oncogene homolog; *EGF* epidermal growth factor; *EGFR* epidermal growth factor receptor; *BNIP3* BCL2/adenovirus E1B 19 kDa interacting protein 3; *PI3* peptidase inhibitor 3; *SP1* Sp1 transcription factor; *STAT3* signal transducer and activator of transcription 3; *LON* Lon protease; *MEF2D* myocyte enhancer factor 2D; *JMY* junction-mediating and regulatory protein, p53 cofactor; *TRAP1* TNF receptor-associated protein 1; *GST* glutathione S-transferase; *NCAM* neural cell adhesion molecule; *CHGA* chromogranin A; *SYP* synaptophysin

### Scoring

The immunohistochemical staining was scored for cytoplasmic and nuclear localization and, when present, for membrane staining. Two observers scored the slides. Intensity was scored on a scale of 0–3 (0 = no staining, 1 = weak, 2 = moderate, 3 = strong staining). The percentage of positive cells was recorded on a scale from 0 to 4 (1 = 1%–10%, 2 = 11%–50%, 3 = 51%–80%, 4 = 81%–100%) or alternatively on a continuous scale from 0% to 100%. The intensity and percentage values were then multiplied to provide a score called the “intensity percentage score” (IPS), the maximum score of which ranged from 12 to 300 [20].

Disputed scores were discussed and a consensus reached. For tissue microarrays, cores that did not contain tumour tissue or that were more than 50% incomplete were excluded. The number of cases scored for each marker was between a minimum of 73 and a maximum of 194 cases for angiogenic tumours and between a minimum of 48 and a maximum of 73 cases for non-angiogenic tumours.

### Statistical analysis

To evaluate the association between protein biomarker expression and angiogenic status, the Mann–Whitney two-tailed non-parametric test was performed using GraphPad Prism statistical analysis software, version 4 (GraphPad Software Inc., San Diego, CA, USA).

### Pathway visualization

Three lists were made: two of proteins more highly expressed in angiogenic or non-angiogenic tumours at least in one subcellular location and the third of proteins always equally expressed in all of their subcellular localizations (Table 2). To visualize the pathways associated with these proteins, each list was imported into the Web-based Enricher facility (http://amp.pharm.mssm.edu/Enrichr/) [21]. The data were visualized from the online databases for gene ontologies (GO biological process and GO cellular component) and for pathways [Kyoto Encyclopedia of Genes and Genomes (KEGG) 2015, WikiPathways 2015, Reactome 2015, and Panther]. The results were visualized by bar graph sorted by combined score.

**Table 2:** Lists of proteins used for pathway visualization using the EnrichrWebd facility [21]

Expression status	Proteins
Equal expression in both tumour types	HIF2, VEGFA, TYMP, KDR, KDRp34, FIH, PHD1, SOD1, EPHB4, BCL2, EGFR, FGF, SP1, LON, MEF2D, RPSA^a^, CHGA, SYP
Higher expression in angiogenic tumours than in non-angiogenic tumours in at least one subcellular location	HIF1, PHD2, PHD3 (cytoplasmic), CXCRN, TSP, DLL4, BNIP3, PI3, EGF, FOS, STAT3, ITGB3^a^, ITGAV^a^
Higher expression in non-angiogenic tumours than in angiogenic tumours in at least one subcellular location	CA9, PHD3 (nuclear), EPHB2, EPHB3, NCAM, P53, TRAP1, JMY, GST

*FGF* fibroblast growth factor; *RPSA* ribosomal protein SA; *ITGB3* integrin, beta 3; *ITGAV* integrin alpha-V; *HIF* hypoxia-inducible factor; *CA9* carbonic anhydrase 9; *VEGFA* vascular endothelial growth factor A; *TYMP* thymidine phosphorylase; *KDR* vascular endothelial growth factor receptor 2; *KDRp34* vascular endothelial growth factor p34; *FIH* factor-inhibiting HIF; *PHD* prolyl hydroxylase dehydrogenase; *DLL4* Delta-like 4; *TSP1* thymidine phosphorylase 1; *CXCR4* chemokine (C-X-C motif) receptor 4; *EPH* ephrin; *SOD1* superoxide dismutase 1; *BCL2* B cell lymphoma 2; *FOS* FBJ murine osteosarcoma viral oncogene homolog; *EGF* epidermal growth factor; *EGFR* epidermal growth factor receptor; *BNIP3* BCL2/adenovirus E1B 19 kDa interacting protein 3; *PI3* peptidase inhibitor 3; *SP1* Sp1 transcription factor; *STAT3* signal transducer and activator of transcription 3; *LON* Lon protease; *MEF2D* myocyte enhancer factor 2D; *JMY* junction-mediating and regulatory protein, p53 cofactor; *TRAP1* TNF receptor-associated protein 1; *GST* glutathione S-transferase; *NCAM* neural cell adhesion molecule; *CHGA* chromogranin A; *SYP* synaptophysin

^a^Data for RPSA are from Ref. [18] and for ITGB3 and ITGAV are from Ref. [23]

### P53 sequencing

Genomic DNA was isolated from 33 specimens of NSCLC (25 angiogenic cases, seven non-angiogenic cases, and one undetermined case) and five specimens of peri-tumour lung tissues. The coding regions corresponding to exons 3–9 (amino acids 25–331) were sequenced. DNA was whole-genome amplified using GenomiPhi (GE Healthcare, Piscataway, NJ, USA). The primers for polymerase chain reaction (PCR) amplification and subsequent sequencing reactions are described in Table 3. PCR was performed using ThermoStart PCR Master Mix (Thermo Fisher Scientific, Waltham, MA, USA), following the manufacturer’s protocol. PCR products were purified and bidirectionally sequenced using the BigDye Terminator cycle sequencing kit, version 1.1 (Applied Biosystems, Foster City, CA, USA), and an ABI 3100 Genetic Analyzer (Applied Biosystem, Paisley, UK). Sequence data were analyzed using Mutation Surveyor, version 3.25 (Softgenetics, State College, PA, USA). Predicted effects on protein were assessed in silico using PolyPhen2 software (http://genetics.bwh.harvard.edu/pph2/). Fisher’s two-sided exact test was performed to compare mutation frequencies in angiogenic versus non-angiogenic cases. All sequencing experiments were performed in duplicate.

**Table 3:** Primers for polymerase chain reaction (PCR) amplification and subsequent P53 sequencing reactions

Primer	Sequence	Annealing temperature (°C)	Amplicon length (bp)
Exon 3–4	Forward: GTGGGAAGCGAAAATTCCAT Reverse: GCCAGGCATTGAAGTCTCAT	60	506
Exon 5–6	Forward: TGTTCACTTGTGCCCTGACT Reverse: TTAACCCCTCCTCCCAGAGA	60	467
Exon 7	Forward: GAGCTTGCAGTGAGCTGAGA Reverse: GGGATGTGATGAGAGGTGGA EX7F_seq CCTGCTTGCCACAGGTCT (to be used for sequencing instead of Exon 7 forward primer)	61.5	390
Exon 8–9	Forward: GACAAGGGTGGTTGGGAGTA Reverse: GCCCCAATTGCAGGTAAAAC	60	500

## Results

### Immunohistochemical staining

The complete results for the cytoplasmic and membranous expression are reported in Table 4, and those for nuclear expression are reported in Table 5, while a summary of these results appears in Table 6. The complete original results of ontology and pathway visualization appear in Additional file 1.

**Table 4:** Cytoplasmic and membranous expression of the proteins in angiogenic versus non-angiogenic non-small cell lung cancers (NSCLCs)

Protein	Intensity percentage score (IPS) of protein expression		*P* value	Tumour type with higher expression
	Angiogenic tumours	Non-angiogenic tumours		
HIF1	6.22 ± 0.33	6.00 ± 0.48	0.87	–
HIF2	0.00 ± 0.00	0.00 ± 0.00	Not applicable	–
CA9 cytoplasm	2.38 ± 0.16	3.50 ± 0.23	<0.001	Non-angiogenic
CA9 membrane	4.00 ± 0.40	3.30 ± 0.88	0.62	–
VEGFA	8.83 ± 0.35	7.38 ± 0.78	0.07	–
TYMP	3.20 ± 0.54	2.21 ± 0.32	0.28	–
KDR	94.56 ± 1.64	97.80 ± 1.54	>0.05	–
KDRp34	10.40 ± 0.30	10.55 ± 0.62	0.71	–
FIH	9.10 ± 0.32	10.00 ± 0.47	0.22	–
PHD1	3.74 ± 0.27	4.20 ± 0.48	0.47	–
PHD2	3.50 ± 0.30	2.46 ± 0.53	0.02	Angiogenic
PHD3	2.70 ± 0.09	3.38 ± 0.17	<0.001	Non-angiogenic
DLL4vessels	2.34 ± 0.10	1.53 ± 0.09	<0.001	Angiogenic
TSP stroma	16.98 ± 2.75	2.00 ± 1.74	<0.001	Angiogenic
CXCR4	2.47 ± 0.20	2.34 ± 0.31	0.92	
EPHB2	7.78 ± 0.27	9.06 ± 0.33	<0.01	Non-angiogenic
EPBH3 cytoplasm	159.3 ± 8.84	202.20 ± 9.95	<0.01	Non-angiogenic
EPBH3 membrane	23.83 ± 7.23	86.51 ± 12.78	<0.001	Non-angiogenic
EPHB4	7.82 ± 0.26	7.40 ± 0.38	0.57	–
SOD1	4.36 ± 0.45	9.39 ± 4.22	0.27	–
BCL2	1.05 ± 0.25	0.68 ± 0.35	0.54	–
FOS	8.05 ± 0.37	5.31 ± 0.55	<0.001	Angiogenic
EGFR	203.40 ± 10.79	166.30 ± 25.74	0.33	–
EGF	18.90 ± 3.03	14.44 ± 4.15	0.72	–
FGF	9.20 ± 0.42	9.72 ± 0.71	0.74	–
BNIP3	6.08 ± 0.36	5.92 ± 0.82	0.88	–
P53	0.28 ± 0.12	2.28 ± 0.56	<0.001	Non-angiogenic
PI3	4.62 ± 0.36	4.92 ± 0.61	0.51	–
SP1	3.70 ± 0.37	3.38 ± 0.55	0.86	–
STAT3	8.78 ± 0.39	7.11 ± 0.79	0.03	Angiogenic
LON	102.50 ± 11.20	134.20 ± 17.10	0.18	–
MEF2D cytoplasm	50.11 ± 4.16	65.67 ± 9.66	0.25	–
MEF2Dmembrane	60.00 ± 7.12	67.50 ± 12.67	0.47	–
JMY	1.84 ± 0.18	2.42 ± 0.31	0.05	Non-angiogenic
TRAP1	4.22 ± 0.28	5.48 ± 0.31	<0.01	Non-angiogenic
GST cytoplasm	236.20 ± 7.16	249.80 ± 11.40	0.23	–
GSTmembrane	28.44 ± 5.52	69.38 ± 14.03	<0.01	Non-angiogenic
NCAM	0.40 ± 0.09	3.92 ± 0.07	<0.001	Non-angiogenic
CHGA	0.08 ± 0.04	0.27 ± 0.14	>0.05	–
SYP	0.12 ± 0.05	0.44 ± 0.15	>0.05	–

All data are presented as mean ± standard error

*HIF* hypoxia-inducible factor; *CA9* carbonic anhydrase 9; *VEGFA* vascular endothelial growth factor A; *TYMP* thymidine phosphorylase; *KDR* vascular endothelial growth factor receptor 2; *KDRp34* vascular endothelial growth factor p34; *FIH* factor-inhibiting HIF; *PHD* prolyl hydroxylase dehydrogenase; *DLL4* Delta-like 4; *TSP1* thymidine phosphorylase 1; *CXCR4* chemokine (C-X-C motif) receptor 4; *EPH* ephrin; *SOD1* superoxide dismutase 1; *BCL2* B cell lymphoma 2; *FOS* FBJ murine osteosarcoma viral oncogene homolog; *EGF* epidermal growth factor; *EGFR* epidermal growth factor receptor; *BNIP3* BCL2/adenovirus E1B 19 kDa interacting protein 3; *PI3* peptidase inhibitor 3; *SP1* Sp1 transcription factor; *STAT3* signal transducer and activator of transcription 3; *LON* Lon protease; *MEF2D* myocyte enhancer factor 2D; *JMY* junction-mediating and regulatory protein, p53 cofactor; *TRAP1* TNF receptor-associated protein 1; *GST* glutathione S-transferase; *NCAM* neural cell adhesion molecule; *CHGA* chromogranin A; *SYP* synaptophysin

*–* Indicates equal expression

**Table 5:** Nuclear expression of the proteins in angiogenic versus non-angiogenic NSCLCs

Protein	Intensity percentage score (IPS) of protein expression		*P* value	Tumour type with higher expression
	Angiogenic tumours	Non-angiogenic tumours		
HIF1	5.49 ± 0.31	3.79 ± 0.39	0.01	Angiogenic
VEGFA	6.09 ± 0.52	5.62 ± 0.32	0.74	–
TYMP	1.94 ± 0.22	1.16 ± 0.21	0.42	–
KDR	231.20 ± 8.55	229.80 ± 9.88	>0.05	–
KDRp34	11.69 ± 0.16	11.45 ± 0.39	0.77	–
FIH	7.50 ± 0.35	8.15 ± 0.72	0.50	–
PHD1	3.10 ± 0.34	2.50 ± 0.35	0.99	–
PHD2	2.56 ± 0.27	1.41 ± 0.35	0.02	Angiogenic
PHD3	2.18 ± 0.13	0.65 ± 0.20	<0.001	Angiogenic
CXCR4	5.13 ± 0.23	3.72 ± 0.37	<0.01	Angiogenic
EPHB2	3.26 ± 0.26	4.91 ± 0.35	<0.001	Non-angiogenic
EPBH3	99.27 ± 7.41	147.30 ± 9.23	<0.001	Non-angiogenic
EPHB4	5.56 ± 0.30	5.73 ± 0.34	0.81	–
SOD1	4.21 ± 0.45	5.00 ± 0.91	0.44	–
C-FOS	7.50 ± 0.52	5.53 ± 0.75	0.05	Angiogenic
EGF	235.20 ± 5.60	214.8 ± 8.68	0.01	Angiogenic
FGF	7.51 ± 0.38	8.68 ± 0.76	0.11	–
BNIP3	3.96 ± 0.40	0.00 ± 0.00	<0.001	Angiogenic
P53	2.66 ± 0.36	2.32 ± 0.53	0.91	–
PI3	0.53 ± 0.13	0.00 ± 0.00	<0.02	Angiogenic
SP1	5.31 ± 0.46	4.04 ± 0.60	0.24	–
STAT3	7.80 ± 0.39	6.31 ± 0.81	0.09	–
MEF2D	251 ± 4.62	243.10 ± 8.04	0.38	–
JMY	2.01 ± 0.17	2.60 ± 0.27	0.07	–
TRAP1	1.57 ± 0.18	4.58 ± 0.32	<0.001	Non-angiogenic
GST	243.30 ± 6.15	244.10 ± 10.91	0.55	–
CHGA	0.00 ± 0.00	0.34 ± 0.12	>0.05	–

All data are presented as mean ± standard error

*HIF* hypoxia-inducible factor; *CA9* carbonic anhydrase 9; *VEGFA* vascular endothelial growth factor A; *TYMP* thymidine phosphorylase; *KDR* vascular endothelial growth factor receptor 2; *KDRp34* vascular endothelial growth factor p34; *FIH* factor-inhibiting HIF; *PHD* prolyl hydroxylasedehydrogenase; *DLL4* Delta-like 4; *TSP1* thymidine phosphorylase 1; *CXCR4* chemokine (C-X-C motif) receptor 4; *EPH* ephrin; *SOD1* superoxide dismutase 1; *BCL2* B cell lymphoma 2; *FOS* FBJ murine osteosarcoma viral oncogene homolog; *EGF* epidermal growth factor; *EGFR* epidermal growth factor receptor; *BNIP3* BCL2/adenovirus E1B 19 kDa interacting protein 3; *PI3* peptidase inhibitor 3; *SP1* Sp1 transcription factor; *STAT3* signal transducer and activator of transcription 3; *LON* Lon protease; *MEF2D* myocyte enhancer factor 2D; *JMY* junction-mediating and regulatory protein, p53 cofactor; *TRAP1* TNF receptor-associated protein 1; *GST* glutathione S-transferase; *NCAM* neural cell adhesion molecule; *CHGA* chromogranin A; *SYP* synaptophysin

*–* Indicates equal expression

**Table 6:** Protein biomarker expression in angiogenic and non-angiogenic NSCLCs according to their intracellular localization

Expression status	Intracellular expression localization	Proteins
Equal expression in both tumour types	Nuclear	VEGFA, TYMP, KDR, KDRp34, FIH, EPHB4, PHD1, SOD1, FGF, P53, SP1, STAT3, MEF2D, JMY, GST, CHGA
	Cytoplasm	HIF1, HIF2, VEGFA, TYMP, KDR, KDRp34, FIH, PHD1, SOD1, CXCR4, EPHB4, BCL2, EGF, EGFR, FGF, BNIP3, PI3, SP1, LON, MEF2D, GST, CHGA, SYP
	Membrane	CA9, MEF2D
Higher expression in angiogenic tumours than in non-angiogenic tumours in at least one subcellular location	Nuclear	HIF1, PHD2, PHD3, CXCR4, BNIP3, PI3, EGF, FOS
	Cytoplasm	PHD2, TSP (stroma), DLL4 (endothelium), FOS, STAT3
	Membrane	None
Higher expression in non-angiogenic tumours than in angiogenic tumours in at least one subcellular location	Nuclear	EPHB2, EPHB3,TRAP1
	Cytoplasm	CA9, PHD3, EPHB2, EPHB3, NCAM, P53, TRAP1, JMY
	Membrane	EPHB3, GST

*HIF* hypoxia-inducible factor; *CA9* carbonic anhydrase 9; *VEGFA* vascular endothelial growth factor A; *TYMP* thymidine phosphorylase; *KDR* vascular endothelial growth factor receptor 2; *KDRp34* vascular endothelial growth factor p34; *FIH* factor-inhibiting HIF; *PHD* prolyl hydroxylase dehydrogenase; *DLL4* Delta-like 4; *TSP1* thymidine phosphorylase 1; *CXCR4* chemokine (C-X-C motif) receptor 4; *EPH* ephrin; *SOD1* superoxide dismutase 1; *BCL2* B cell lymphoma 2; *FOS* FBJ murine osteosarcoma viral oncogene homolog; *EGF* epidermal growth factor; *EGFR* epidermal growth factor receptor; *BNIP3* BCL2/adenovirus E1B 19 kDa interacting protein 3; *PI3* peptidase inhibitor 3; *SP1* Sp1 transcription factor; *STAT3* signal transducer and activator of transcription 3; *LON* Lon protease; *MEF2D* myocyte enhancer factor 2D; *JMY* junction-mediating and regulatory protein, p53 cofactor; *TRAP1* TNF receptor-associated protein 1; *GST* glutathione S-transferase; *NCAM* neural cell adhesion molecule; *CHGA* chromogranin A; *SYP* synaptophysin

In Table 7, a selection of pathways shows that proteins expressed in both types of tumours are associated with angiogenesis-, VEGF-, and oxidative stress-related pathways. The non-angiogenic tumours are distinguished from the angiogenic tumours by having higher levels of proteins related to ephrin pathways, response to hypoxia, HIF1 regulation by oxygen, and transcription of HIF-controlled genes but lower levels of proteins involved in stromal cell–cell signaling and adhesion, integrins, and Delta-Notch- and EGF-related signaling.

**Table 7:** Selection of visualized pathways

Proteins equally expressed in angiogenic and non-angiogenictumours		Proteins up-regulated in angiogenic tumours		Proteins up-regulated in non-angiogenic tumours	
Pathway	Databases	Pathway	Databases	Pathway	Databases
Focal adhesion	KEGG, WikiPathways	Focal adhesion	KEGG, WikiPathways	–	–
VEGF-related	KEGG, Panther, Reactome	–	–	–	–
Angiogenesis	Panther, WikiPathways	Angiogenesis	Panther	Angiogenesis	Panther
–	–	Itegrin signalling	Panther	–	–
Oxidative stress	Panther, WikiPathways	–	–	–	–
Cell response to stress	Reactome	–	–	–	–
–	–	Delta-Notch	WikiPathways	–	–
–	–	Collagen-related pathways	Reactome	–	–
–	–	Cell–cell communication	Reactome	–	–
–	–	Integrin signaling	Panther	–	–
		EGF-related pathways	WikiPathways, Panther	–	–
–	–	–	–	Response to Hypoxia	Reactome
Ephrins signaling	Reactome	–	–	Ephrins-related pathways	Reactome
–	–	–	–	Regulation of HIF by oxygen	Reactome
–	–	–	–	Regulation of genes by HIF	Reactome
–	–	–	–	Nitrogen metabolism	KEGG

Gene ontology analysis confirmed that proteins usually associated with angiogensis were present in both types of tumours, whereas higher levels of proteins associated with Notch, extracellular matrix, and cell adhesion were present in the truly angiogenesis-producing tumours. However, in the tumours that grow in a non-angiogenic fashion, proteins associated with mitochondria, cell biogenesis, carbonic dehydratase, ephrins, and axon guidance-related functions were more commonly detected (Table 8).

**Table 8:** Selection of visualized ontologies

Proteins equally expressed in angiogenic and non-angiogenic tumours		Proteins up-regulated in angiogenic tumours		Proteins up-regulated in non-angiogenic tumours	
Ontology	GO-ontology database	Ontology	GO-ontology database	Ontology	GO-ontology database
Cell migration/sprouting angiogenesis GO.0002042	Biological process	Immune response-activating signals GO.0002757	Biological process	Regulation of synapses GO.0051965	Biological process
Endothelial cell migration GO.0043534	Biological process	Immune response-regulating cell signaling GO.0002768	Biological process	Axon guidance and neuronal regulation GO.0031290 GO.0007413 GO.0021952 GO.0021955 GO.0008038	Biological process
VEGF signaling pathways GO.0038084	Biological process	Mesodermal cell differentiation GO.0048333	Biological process	–	–
–	–	Activation of immune response GO.0002253	Biological process	–	–
–	–	–	–	Positive regulation cell biogenesis GO.0044089	Biological process
–	–	Basement membrane GO.0005604	Cell component	–	–
–	–	Extracellular matrix part GO.0044420	Cell component	Mitochondrial intermembrane space and matrix GO.0005758 GO.0005759	Cell component
–	–	Complex involve in cell adhesion GO.0098636	Cell component	–	–
PDGF receptor-binding GO.0005161	Molecular function	Fibronectin- and extracellular matrix-binding GO.0001968 GO.0050840	Molecular function	Ephrin receptor activity GO.0005003	Molecular function
Extracellular matrix-binding GO.0050840	Molecular function	Notch-binding GO.0005112	Molecular function	Carbonate dehydratase activity GO.0004089	Molecular function
–	–	–	–	Axon guidance GO.0008046	Molecular function

### Sequencing

The higher level of *p53* expression in the cytoplasm of the non-angiogenic tumours, as compared with the angiogenic tumours, was one of the most striking results (Table 9) and raised the question of whether non-angiogenic cells had wild-type *p53* or a different set of mutations from the angiogenic tumours. Our results showed that *p53*mutations were observed in eight of 25 angiogenic cases (32.0%) but in five of seven non-angiogenic cases (71.4%) (*P* = 0.091 by Fisher’s two-tailed exact test) (Table 9).

**Table 9:** Summary of *p53* mutations detected in non-angiogenic and angiogenic NSCLC cases

Sample ID	Sample type	Mutation location	Mutation type	Domain	Predicted effect on protein activity
104	Non-angiogenic	c.761T>TA; p.I254S	Missense	HCD IV	Damaging
105	Non-angiogenic	c.734G>GA; p.G245D	Missense	HCD IV	Damaging
121	Non-angiogenic	c.488A>AG; p.Y163C	Missense	DNA binding	Damaging
152	Non-angiogenic	c.634T>TG; p.F212 V	Missense	DNA binding	Benign
249	Non-angiogenic	c.314G>GA; p.G105D	Missense	DNA binding	Damaging
133	Angiogenic	c.511G>GT; p.E171X	Nonsense (truncated protein)	HCD III	Truncating
138	Angiogenic	c.824G>GA; p.C275Y	Missense	HCD IV	Damaging
139	Angiogenic	c.het_del216C; p.V73 Wfs48X	Frameshift (truncated protein)	Prolinerich	Truncating
141	Angiogenic	c.407A>AC; p.Q136P	Missense	HCD II	Damaging
98	Angiogenic	c.524G>GA; p.R175H	Missense	HCD III	Potentially damaging
147	Angiogenic	c.524G>GA; p.R175H	Missense		
274	Angiogenic	c.471_472TC>GA; p.V157G	Missense	DNA binding	Damaging

Affected domains are listed. The software used to predict the functional effect of the detected sequence changes was PolyPhen_2 (http://genetics.bwh.harvard.edu/ggi/pph2/c2ea64efde6f039a5ca76a2a264ae4f3cf922360/1121012.html )

*HCD* highly conserved domain

**Fig. 1:**
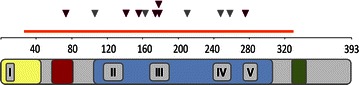
Localization of the mutations detected on the *p53* gene. The sequenced region is indicated with an *orange* line in the figure above. Mutation locations are indicated with *arrowheads* (*purple* mutations found in angiogenic samples; *gray*: mutations found in non-angiogenic samples). Human P53 protein (HP53) can be divided into five domains, each corresponding to specific functions: *yellow* is the highly conserved domain I (HCD I)/transactivation domain; *red* is the second transactivation domain, which is proline-rich; *blue* is the DNA-binding domain essential for *p53*-DNA interactions that also contains HCD II-V and is the target of 90% of the *p53* mutations found in human cancers; *green* is the nuclear export signal (NES) localized in the oligomerization domain of *p53*

All of the detected mutations were heterozygous, and almost all of them corresponded to hot spots previously reported in different tumour types (http://genetics.bwh.harvard.edu/ggi/pph2/c2ea64efde6f039a5ca76a2a264ae4f3cf922360/1121012.html). The locations of the mutations were randomly distributed across the sequenced region. No specific pattern of mutation location seemed to be related to tumour subtype, angiogenic or non-angiogenic (Fig. 1 and Table 9).

## Discussion

As initially suggested by our mRNA profiling work [19] and also confirmed by the immunohistochemical data presented here, we failed to reveal significant differences between angiogenic and non-angiogenic NSCLCs as far as the expression of proteins associated with the classic hypoxia/angiogenesis pathway is concerned. Because neovascularisation is found in some tumours but not in others, we suggest that activation of the classical angiogenic pathways is necessary, but not sufficient, to induce the sprouting of new vessels in cancer.

The higher levels of expression of proteins associated with extracellular matrix, cell adhesion, and inflammation were in agreement with the observed presence of tumour-associated stroma and chronic inflammation in many angiogenic tumours [18, 22]. mRNA profiling of these tumours has equally shown that stromal remodeling, cell adhesion, and inflammation are enhanced in angiogenesis [19]. Both mRNA and immunohistochemical data suggest a crucial role in angiogenic cancers for FBJ murine osteosarcoma viral oncogene homolog (FOS), a protein involved in cell proliferation, remodeling, and inflammation. The question remains of whether tissue remodeling is a consequence or a cause of the triggering of angiogenesis. Because non-angiogenic tumours usually preserve the pre-existing architecture, we hypothesize that the triggering of tissue destruction could be a secondary step necessary for the activation of angiogenesis. Nonetheless, this process would be far from a general rule because we have observed, in a subset of NSCLC, that angiogenesis can occur in the absence of tissue destruction [23]. Clearly, other mechanisms must exist.

If classical angiogenesis pathways are similarly active in both angiogenic and non-angiogenic tumours, how does their biology differ? Our previous transcriptional profiling work demonstrated higher levels of mRNA coding for molecules associated with oxidative phosphorylation and mitochondrial biogenesis in non-angiogenic tumours [19]. In the present study, we did not examine components of the oxidative phosphorylation pathway, although we did investigate the expression of some proteins involved in mitochondrial functions. Our results showed that proteins related to mitochondria and to cell biogenesis-promoting processes were more highly expressed in non-angiogenic tumours than in angiogenic tumours. This feature was consistent with higher mitochondrial regulatory activity and a possible metabolic switch in non-angiogenic tumours. Again, whether this switch is a cause or an effect of HIF activation remains unclear.

Interestingly, our data suggested increased involvement of response to hypoxia and to HIF regulation by oxygen in non-angiogenic tumours. We hence speculate that, in these tumours, although the HIF pathway failed to induce new vessel formation, it could well be involved in metabolic reprogramming.

Finally, we noted markedly higher levels of cytoplasmic P53 expression in non-angiogenic tumours than in angiogenic tumours via immunohistochemistry. In a pilot study, we sequenced *p53* in a limited number of angiogenic and non-angiogenic NSCLCs. Non-angiogenic tumours had a higher incidence of mutations, which were all missense mutations, whereas angiogenic tumours had an amalgam of frame shifts and missense and nonsense mutations. Because *p53* also affects mitochondrial respiration [24], it will be necessary to investigate further how the observed *p53* mutations could functionally affect its ability to regulate respiration and/or angiogenesis.

## Conclusions

On the basis of our observations collected so far, from the mRNA profiling, immunohistochemical and histopathologic data, we conclude that all tumours, angiogenic and non-angiogenic, experience hypoxia/HIF and VEGF pathway activation. However, angiogenesis does not always ensue. Based on these findings, we suggest that in non-angiogenic tumours, HIF pathway activation could be the driving force toward metabolic reprogramming.

## Additional file

10.1186/s40880-016-0082-6 Visualization of ontologies and pathways likely to be associated with the patterns of protein expression as detected by immunohistochemistry on angiogenic versus non-angiogenic non-small cell lung carcinomas (NSCLCs). The results represent the combined score, which is computed by the Enrich application by taking the log of the *P* value from the Fisher’s exact test and multiplying it by the *z* score of the deviation from the expected rank. The length of the bar represents the significance of that specific geneset or term. In addition, the brighter the color is, the more significant that term is. The following are the original results classified according to the databases used (KEGG 2015, Panther, Reactome 2015, WikiPathways 2015, GO-Biological Process, GO-Cell Component, and GO-Molecular Function).
