# Supplementary material for: Why some tumours trigger neovascularisation and others don’t: the story thus far
Source: Chin J Cancer. 2016 Feb 12;35:18. doi: 10.1186/s40880-016-0082-6 (PMC4752802; doi:10.1186/s40880-016-0082-6)

**Supplementary Figure S1.Visualization of ontologies and pathways likely to be associated with the patterns of protein expression as detected by immunohistochemistry on angiogenic versus non-angiogenic non-small cell lung carcinomas (NSCLCs).** The results represent the combined score, which is computed by the Enrich application by taking the log of the *P* value from the Fisher’s exact test and multiplying it by the *z* score of the deviation from the expected rank. The length of the bar represents the significance of that specific geneset or term. In addition, the brighter the color is, the more significant that term is. The following are the original results classified according to the databases used (KEGG 2015, Panther, Reactome 2015, WikiPathways 2015, GO-Biological Process, GO-Cell Component, and GO-Molecular Function).

Proteins with comparable expression in angiogenic and non-angiogenic NSCLC.

KEGG 2015


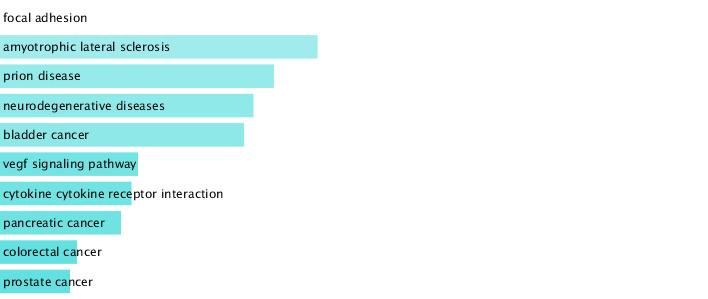


Panther


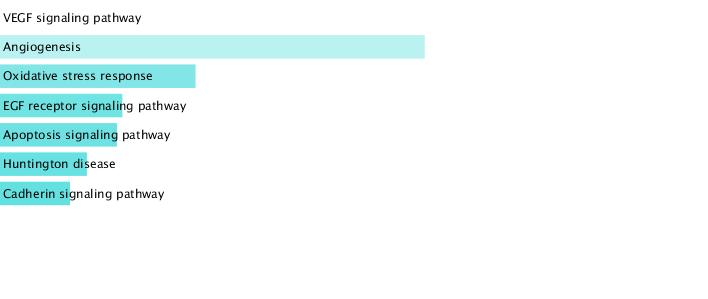


Reactome 2015


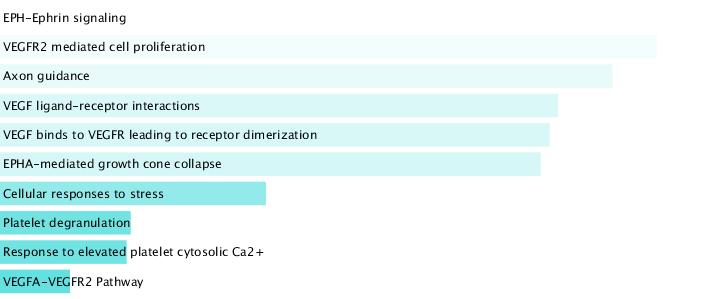


WikiPathways 2015

**
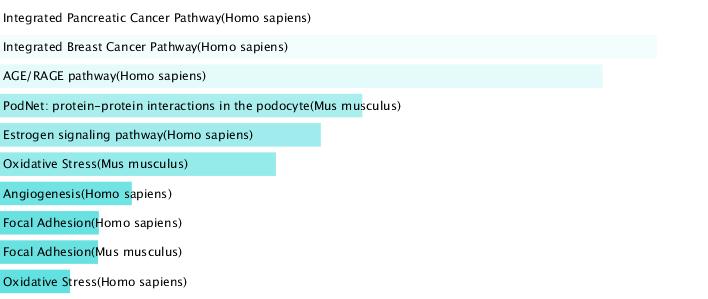
**

GO-Biological Process


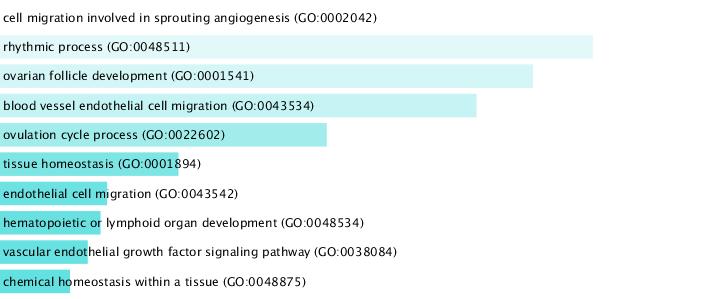


GO-Cellular Component


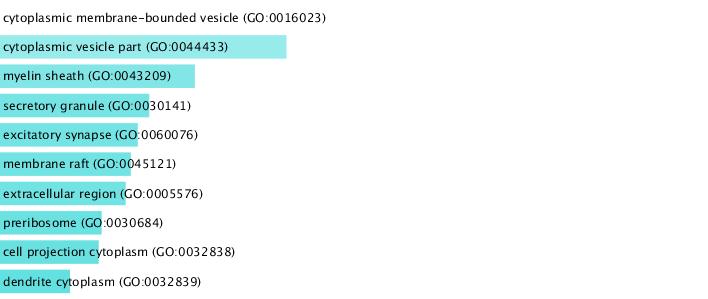


GO-Molecular Function


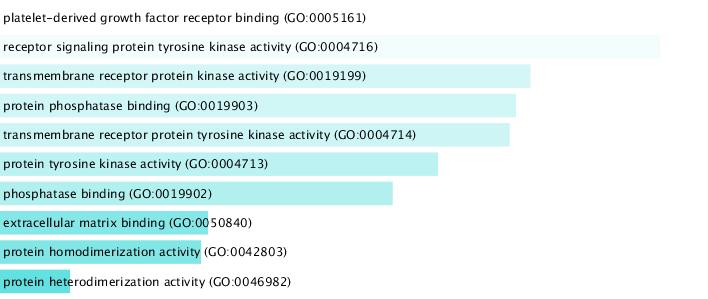


Proteins with higher expression in AngiogenicNSCLC.

KEGG 2015


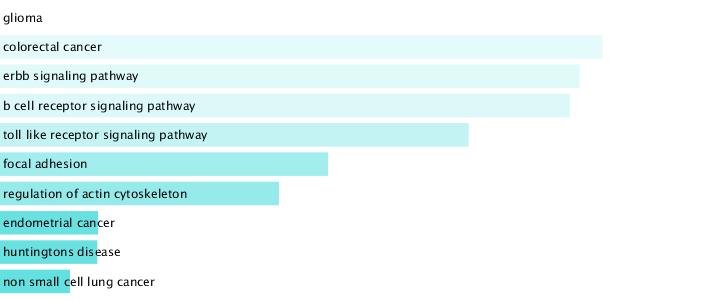


PANTHER


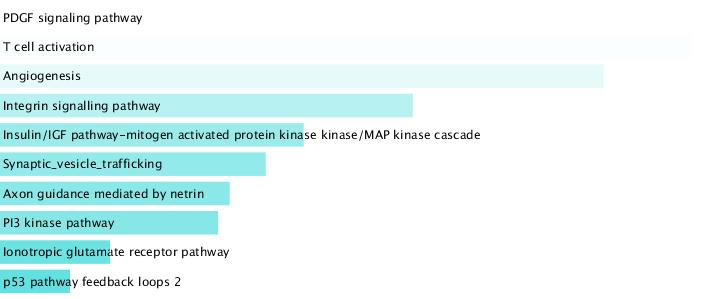


Reactome 2015


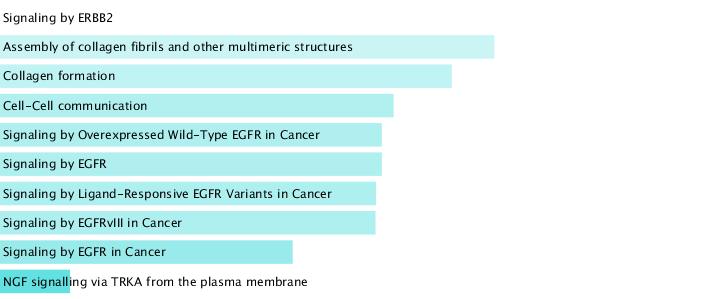


WikiPathways 2015


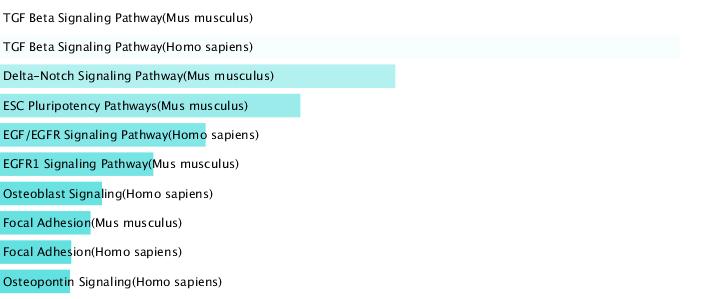


GO-Biological process


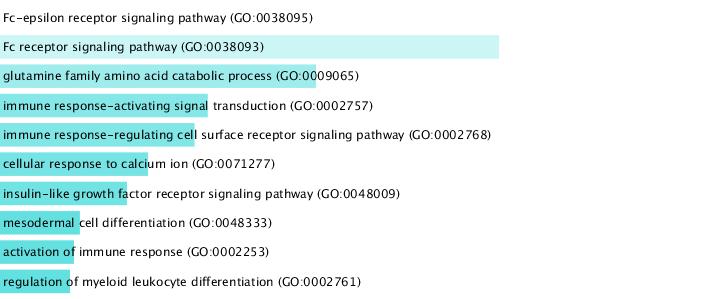


GO-Cell Component


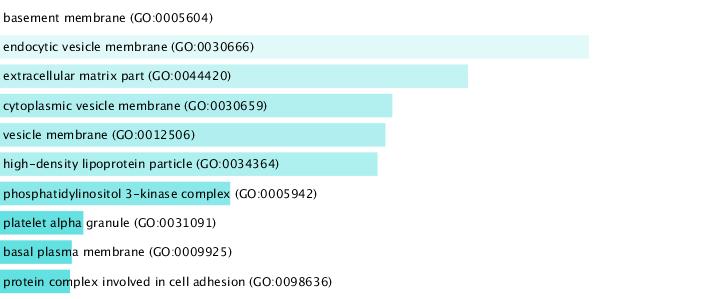


GO-Molecular Function


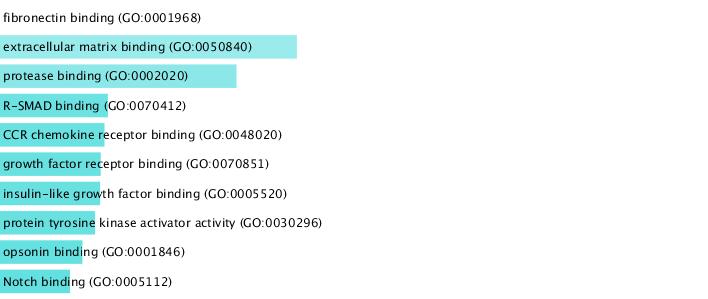


Proteins with higher expression in Non-Angiogenic NSCLC.

KEGG 2015


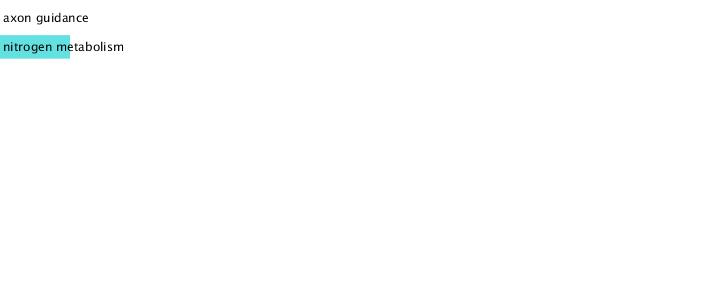


Panther


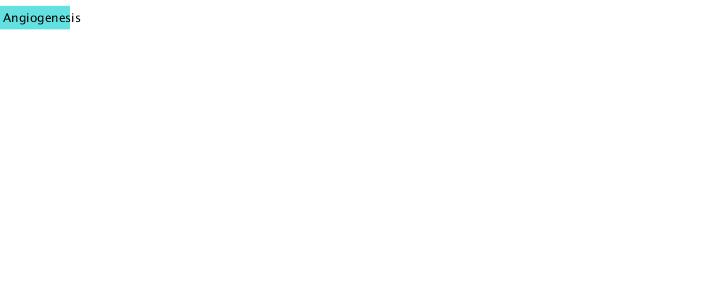


Reactome 2015
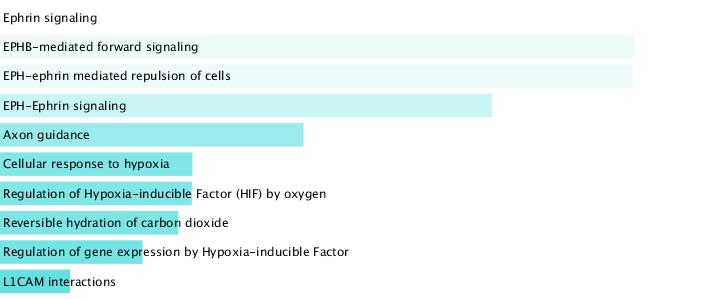


WikiPathways 2015


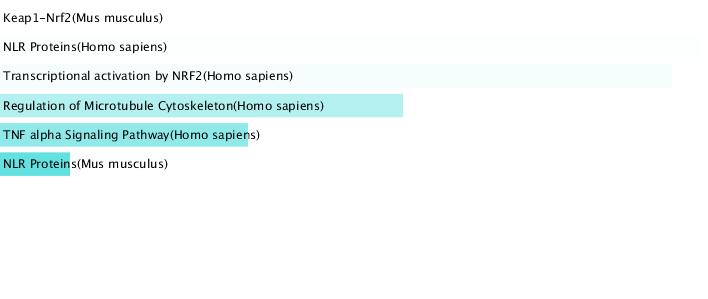


Go-Biological Process


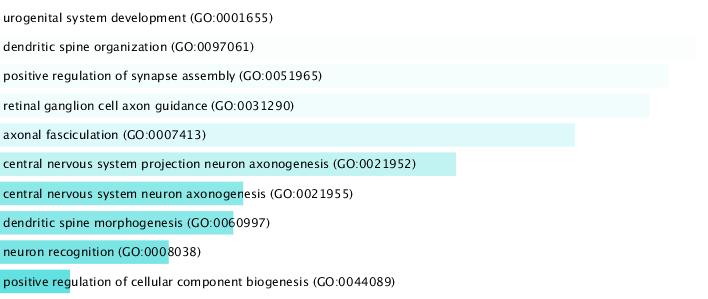


Go-Cellular Component


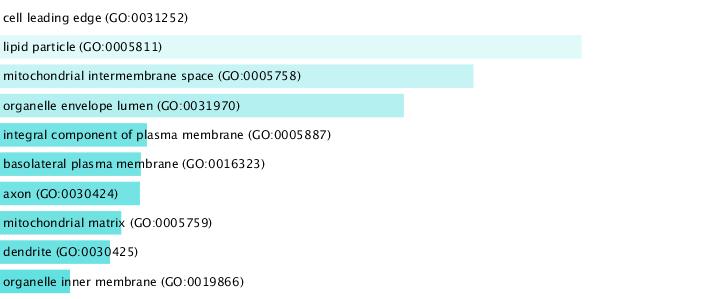


GO-molecular function


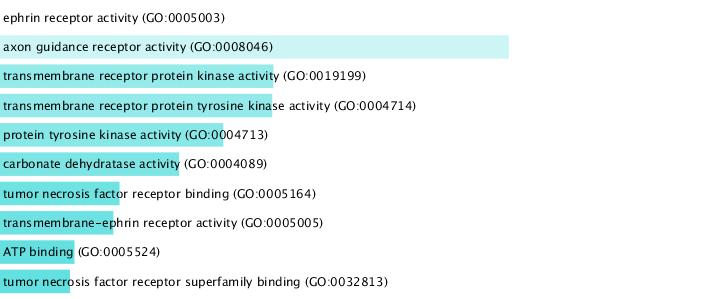

Supplement: Supplementary file 1 — 10.1186/s40880-016-0082-6 Visualization of ontologies and pathways likely to be associated with the patterns of protein expression as detected by immunohistochemistry on angiogenic versus non-angiogenic non-small cell lung carcinomas (NSCLCs). The results represent the combined score, which is computed by the Enrich application by taking the log of the P value from the Fisher’s exact test and multiplying it by the z score of the deviation from the expected rank. The length of the bar represents the significance of that specific geneset or term. In addition, the brighter the color is, the more significant that term is. The following are the original results classified according to the databases used (KEGG 2015, Panther, Reactome 2015, WikiPathways 2015, GO-Biological Process, GO-Cell Component, and GO-Molecular Function). [file 40880_2016_82_MOESM1_ESM.docx]
